# New Biomarkers of Coffee Consumption Identified by the Non-Targeted Metabolomic Profiling of Cohort Study Subjects

**DOI:** 10.1371/journal.pone.0093474

**Published:** 2014-04-08

**Authors:** Joseph A. Rothwell, Yoann Fillâtre, Jean-François Martin, Bernard Lyan, Estelle Pujos-Guillot, Leopold Fezeu, Serge Hercberg, Blandine Comte, Pilar Galan, Mathilde Touvier, Claudine Manach

**Affiliations:** 1 INRA, UMR 1019, Human Nutrition Unit, CRNH Auvergne, Clermont-Ferrand, France; 2 Clermont University, Human Nutrition Unit, Clermont-Ferrand, France; 3 INRA, Plateforme d'Exploration du Métabolisme, Clermont-Ferrand, France; 4 Paris 13 University, Sorbonne Paris Cité, Nutritional Epidemiology Research Team, Epidemiology and biostatistics Research Center, INSERM U1153, INRA U1125, CNAM, Paris 5 University, Paris 7 University, Bobigny, France; Kobe University, Japan

## Abstract

Coffee contains various bioactives implicated with human health and disease risk. To accurately assess the effects of overall consumption upon health and disease, individual intake must be measured in large epidemiological studies. Metabolomics has emerged as a powerful approach to discover biomarkers of intake for a large range of foods. Here we report the profiling of the urinary metabolome of cohort study subjects to search for new biomarkers of coffee intake. Using repeated 24-hour dietary records and a food frequency questionnaire, 20 high coffee consumers (183–540 mL/d) and 19 low consumers were selected from the French SU.VI.MAX2 cohort. Morning spot urine samples from each subject were profiled by high-resolution mass spectrometry. Partial least-square discriminant analysis of multidimensional liquid chromatography-mass spectrometry data clearly distinguished high consumers from low via 132 significant (p-value<0.05) discriminating features. Ion clusters whose intensities were most elevated in the high consumers were annotated using online and in-house databases and their identities checked using commercial standards and MS-MS fragmentation. The best discriminants, and thus potential markers of coffee consumption, were the glucuronide of the diterpenoid atractyligenin, the diketopiperazine cyclo(isoleucyl-prolyl), and the alkaloid trigonelline. Some caffeine metabolites, such as 1-methylxanthine, were also among the discriminants, however caffeine may be consumed from other sources and its metabolism is subject to inter-individual variation. Receiver operating characteristics curve analysis showed that the biomarkers identified could be used effectively in combination for increased sensitivity and specificity. Once validated in other cohorts or intervention studies, these specific single or combined biomarkers will become a valuable alternative to assessment of coffee intake by dietary survey and finally lead to a better understanding of the health implications of coffee consumption.

## Introduction

Coffee is one of the most widely consumed beverages in the world. It is consumed on a daily basis in most of the United States, Canada, Japan, New Zealand, and in Europe, where consumption is greatest in Scandanavian countries [1]. Due to its rich phytochemistry and frequent consumption, the beverage has a complex relationship with human health, and may be responsible for both negative and positive health effects [2], [3]. Coffee intake is known to increase blood pressure [4], [5] and when consumed in excess may also elevate the risk of cardiovascular disease [6]. Consumption has also recently been associated with increased all-cause mortality [7]. In contrast, recent epidemiological studies have suggested that regular coffee consumption could decrease the risk of type II diabetes, Parkinsonism, Alzheimer's disease, liver cancer, and even the risk of stroke [2], [8]–[10].

Coffee contains several bioactives of potential importance to human health. Firstly, it is the major dietary source of the alkaloid stimulant caffeine, long believed to influence vascular health. Secondly, the beverage is rich in phenolic acids, particularly isomers of caffeoylquinic acid, the greatest contributor to polyphenol intake in European populations [11], [12]. Thirdly, coffee contains a range of other potential bioactives whose metabolites may influence human health at lower concentrations. For example, coffee diterpenoids cafestol and kahweol may be chemopreventive but also raise cholesterol levels in healthy humans [13]. Risks and benefits may vary between individuals, depending on individual risk factors for the diseases, genetic variation affecting caffeine metabolism and pharmacodynamics, background diet, and method of coffee preparation. The accurate assessment of coffee consumption in observational studies, as with other foods of dietary importance, is problematic. In the large-scale epidemiological studies needed to characterize the links between diet and health, intake data are collected by dietary questionnaires, which rely on accurate self-reporting by participants. Despite advances in data collection techniques, bias remain a problem, and burdensome and expensive protocols using multiple 24 h dietary recalls and/or validated thorough food frequency questionnaires (FFQs) are necessary to obtain accurate assessment of food intake.

A potential alternative to these assessment methods is the use of biomarkers, which if well-validated could reliably reflect the recent and/or habitual consumption of a food or dietary pattern of interest [14]. Until recently, these could only be discovered *ad-hoc* based on knowledge of the metabolism of certain food components [15]. Metabolome profiling now allows the more efficient discovery of biomarkers of intake. Biofluids from low and high consumers or intervention and control subjects are profiled and compared, and the signals responsible for the variation investigated as potential biomarkers [16]. Several biomarkers have been proposed for coffee intake from intervention studies but none has been evaluated yet in a large cohort study. The aim of the present study was to search for reliable biomarkers of coffee intake by profiling the biobanked urines of SU.VI.MAX2 cohort subjects who had reported either high or low habitual consumption. The use of samples from cohort studies rather than controlled interventions have allowed us to propose new candidate biomarkers that are robust and stable enough to be measured in any type of study, as recently demonstrated for the intake of citrus fruit [16].

## Materials and Methods

### Standards and reagents

Standards of caffeine, 1-methyluric acid, trigonelline (1-methylpyridinium-3-carboxylate) hydrochloride, hippuric acid, theophylline and paraxanthine were purchased from Sigma-Aldrich (L'Isle d'Abeau, France). 1,7-dimethyluric acid and 5-acetylamino-6-formylamino-3-methyluracil (AFMU) were supplied by Toronto Research Chemicals Inc (Canada). Cyclo(leucyl-prolyl) was obtained from Bachem (USA) and 3-hydroxyhippuric acid was kindly provided by P.C.H. Hollman (Rikilt, Wageningen Univ., The Netherlands). For urine hydrolysis, *β*-Glucuronidase - sulfatase from *Helix pomatia* (G0786) was purchased from Sigma-Aldrich (France). HPLC-grade acetonitrile and formic acid were supplied by Sigma-Aldrich (France) and water used for liquid chromatography-mass spectrometry (LC-MS) was generated from a Milli-Q integral water purification system.

### Subjects

Study subjects were participants in the *SUpplémentation en VItamines Mineraux et AntioXydants 2* (SU.VI.MAX 2) cohort. SU.VI.MAX and SU.VI.MAX 2 followed 13,000 and 6850 participants respectively between 1994 and 2009 to investigate the effect of nutrition on the quality of aging [17]. The study was conducted according to the guidelines of the Declaration of Helsinki and approved by the Ethical Committee for Studies with Human Subjects of Paris-Cochin Hospital (CCPPRB N° 706 and 2364, respectively) and the *Comité National Informatique et Liberté* (N° 334641 and 907094, respectively). All participants signed an informed consent form approved by the Ethical Committee. Every two months, participants were invited to complete a 24 h dietary record via the Minitel Telematic Network, a French telephone-based terminal equivalent to an Internet prototype widely used at the beginning of the study. The records were obtained between weeks and weekends in a 2∶1 ratio and evenly between seasons to take into account intra-individual variability. Participants assessed portion sizes using a validated picture booklet. In 2009, the SU.VI.MAX2 subjects also completed a validated food frequency questionnaire (FFQ) [18]. For the PhenoMeNEp study (Phenotyping using Metabolomics for Nutritional Epidemiology), 144 high and 66 low consumers of fruit and vegetables (F&V), balanced for age, gender, BMI, and season of sampling, were randomly selected from the highest and lowest quartiles of F&V consumption as reported both in six detailed 24 h records and one FFQ obtained from the cohort between 1998 and 2009. In anticipation of validating the statistical models externally, 50 additional subjects were selected from all quartiles of F&V consumption. For the present study, whose aim was to identify biomarkers of coffee intake, subgroups of 19 low and 20 high coffee consumers were further selected from the 210 SU.VI.MAX2 subjects. These were all subjects who had reported either high (>180 mL/d) or no coffee consumption consistently in the dietary records and in the FFQ. The selection was based on the distribution of coffee intake, taking into account the correlation of consumption of other foods such as chocolate or red wine, as shown in Supporting Information S1.

### Profiling of urine samples

For each of the 260 PhenoMeNEp subjects, one morning spot urine from the SU.VI.MAX2 biobank (collected between 2007 and 2009) was profiled using UPLC-QTof-MS. Urine samples were centrifuged at 12,000 g for 4 min and diluted two-fold with Milli-Q purified water. Samples were profiled in a randomized sequence using a Waters Acquity UPLC module (Waters, Manchester, UK) coupled to a Waters QToF-Micro mass spectrometer equipped with an electrospray source operated both in positive (ESI+) and negative (ESI−) ionization modes, and a lock-mass sprayer to ensure accuracy. Details of the analysis conditions have been previously published [16].

### Data pre-processing and statistical analysis

Full-scan UPLC-QTof-MS data for the 260 subjects were extracted and processed using XCMS software [19]. To correct for drift between different series, ion intensities were normalized using data from quality controls consisting of pooled study urine samples [20]. The datasets obtained for positive and negative modes were merged and then filtered to remove any ions that did not appear in more than 25% of the samples of at least one group (low or high F&V consumers). For the present study, statistical analysis was then carried out on a reduced dataset comprising the data matrix of the 19 low- and 20 high coffee consumers. Data were log transformed and an orthogonal signal correction (OSC) [21] filter with Pareto scaling was applied to all remaining ions to reduce variability not associated with the diet effect. Both univariate and multivariate statistical approaches were used. On the OSC filtered dataset, a one-way ANOVA (R software) was performed on each ion to search for differences in intensities between the selected low and high coffee consumers, applying a Benjamini-Hochberg (BH) [22] p-value correction to reduce the risk of false positive discovery. All ions with p-value<0.05 were considered statistically significant. In parallel, a partial least squares discriminant analysis (PLS-DA) was performed on all detected ions (SIMCA-P+ software, version 13.0, Umetrics AB, Umea, Sweden). The predictive ability of the PLS model was assessed by cross validation (Q^2^cum) and permutation test (n = 100; plot and CV ANOVA). Variable Importance Projection (VIP) values were obtained as indicators of importance of each ion in the discrimination.

Receiver Operating Characteristics (ROC) curves, widely considered to be the most objective and statistically valid method for the evaluation of biomarker performance, were constructed using the ROCCET web-based tool [23]. As with the previous statistical analysis, data used for ROC analyses were those filtered with OSC. The 20 high and 19 low-coffee consumers selected for the study were used as the training set and 10 high and 10 low-coffee consumers were selected among the validation population as the hold-out set. Both individual and multiple biomarker ROC curves were built using the support vector machines algorithm. The average of predicted class probabilities of each sample and the average predicted accuracy were then calculated across 100 cross-validations, giving the respective confusion matrices for the training and hold-out sets.

The Pearson's correlation coefficient between the intensity of some discriminating ions and the declared coffee consumption was determined in the SU.VI.MAX2 sub-population of 260 subjects selected for the PhenoMeNEp project.

### Identification of discriminant ions

Ions with ANOVA p-value less than 0.05, VIP greater than 1 and a mean intensity ratio high consumers/low consumers greater than 1 (OSC filtered values) were retained for further investigation. The list of discriminants was first grouped into clusters according to LC retention time (RT), and correlation between fragments was confirmed by a visual check of the corresponding extracted ion chromatograms (EIC) of the UPLC-QTof analysis and comparison of their distribution across the whole dataset.

To obtain accurate masses, molecular formulae, and additional structural information, a small number of representative samples were also analyzed using a high resolution LTQ Orbitrap Velos™ hybrid mass spectrometer (Thermo Fisher Scientific, San José, CA) operating in various modes (i.e. full scan and collision induced dissociation fragmentation) using mass resolution from 7500 to 30000. Chromatographic separation was achieved with a RSLC Ultimate 3000 liquid chromatography module (Dionex), using the same conditions as for the UPLC-QTof-MS analysis.

The ions of interest were retrieved in the LTQ-Orbitrap full scan chromatograms to obtain accurate masses for these signals, which generally allowed the determination of molecular formulae. Online databases such as the Human Metabolome Database (HMDB; www.hmdb.ca), KNApSAcK (http://kanaya.naist.jp/knapsack_jsp/top.html), Dictionary of Natural Products (http://dnp.chemnetbase.com) and Metlin (http://metlin.scripps.edu) were queried to hypothesize identities. In addition, a customized in-house database on coffee phytochemical metabolites was used, comprising all phytochemicals reported in coffee according to Phenol-Explorer (http://www.phenol-explorer.eu), Duke's Phytochemical and Ethnobotanical Database (http://www.ars-grin.gov/duke), the Dictionary of Natural Products, KNApSAcK and a literature survey, as well as their known metabolites reported in the literature. Additional likely human metabolites of coffee constituents were also included, as predicted *in silico* by the software Meteor-Nexus (v.13.0.0, Lhasa Ltd, Leeds, UK). If a hypothesis was within 1 mDa of a chemically and biologically plausible coffee-derived metabolite, the commercial standard was acquired to perform the final confirmation of identity. If this was not available, fragmentation was performed on the LTQ-Orbitrap and the resulting spectra examined and compared to those available in online databases or literature. *In silico* prediction of the mass fragmentation of the candidate structures was also performed using Mass Frontier™ software (Thermo Fisher Scientific, San José, CA). The characterization of sulfated and glucuronidated derivatives was carried out by comparing profiles obtained before and after enzymatic hydrolysis of urine samples. For hydrolysis, urine samples (100 µl) were incubated overnight in sodium acetate buffer at pH 4.9 in the presence of β-glucuronidase (1000 U) and sulfatase (45 U).

## Results

The 20 high coffee consumers reported a median intake of 290 mL/d (range: 183–540 mL/d), whereas all low consumers reported zero habitual consumption in all questionnaires. No significant differences in sex, age, season of urine sampling or BMI were observed between the two groups (Chi-squared p-value = 0.408, 0.886, 0.069 and 0.869, respectively).

**Figure 1:**
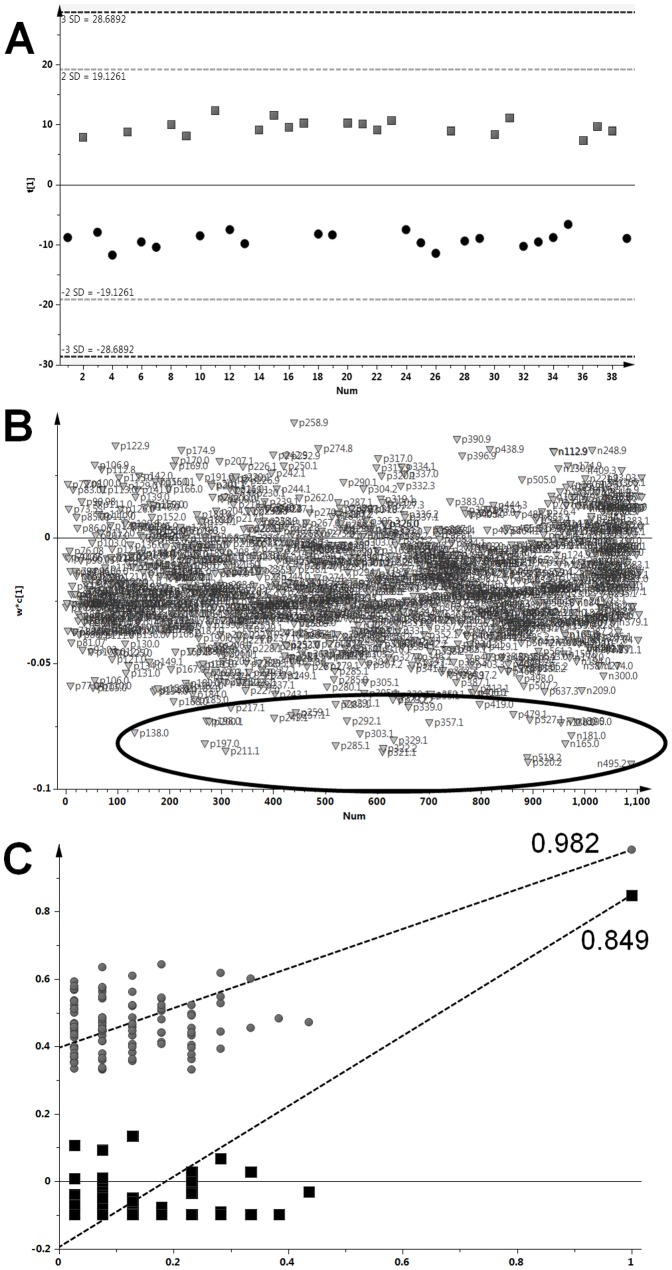
Metabolomic profiling of spot urines from SU.VI.MAX2 subjects. Subjects reported either low or high consumption of coffee, represented by squares and circles respectively. A) One-dimensional OSC-PLS-DA score plot of urinary metabolomes of low and high consumers. B) Loading plot of the OSC-PLS-DA. Circled outlying ions contribute most strongly to the discrimination. C) Model validation assessed by permutation test (*n* = 100).

After data pre-processing, 932 and 179 ions were detected in urine analyzed in positive and negative modes, respectively. Data were compared using both univariate and multivariate statistics. ANOVA with false positive discovery BH correction showed that 119 and 13 ions in positive and negative modes respectively had significantly different intensities (p-value<0.05) in the groups of low and high coffee consumers. All significant ions except one (*m/z* 258.905) had higher intensities in the group of high consumers than in the low, suggesting an exogenous origin for the majority of the significant ions. In parallel, the OSC-PLS-DA of the urine metabolomic profiles comprising all 1111 detected ions clearly distinguished the groups of low and high coffee consumers (**Figure 1A**). The calculated cumulative ratio Q^2^(cum) (0.849) combined with a low CV-ANOVA (1.77×10^−15^) and a good permutation test plot (Figure 1C) indicated an excellent validation of the PLS model. This multivariate analysis revealed that 360 ions contributed to the discrimination (VIP>1), of which 47 were particularly resonant (VIP>2). Use of ANOVA BH p-values and the OSC-PLS-DA VIP values notably gave similar ion rankings, and all the 132 ions with ANOVA BH p-value<0.05 also had a VIP value>1.5. The 132 ions corresponded to around 60 metabolites detected as clusters of correlated ions comprising fragments and adducts and sharing the same retention time.

**Figure 2:**
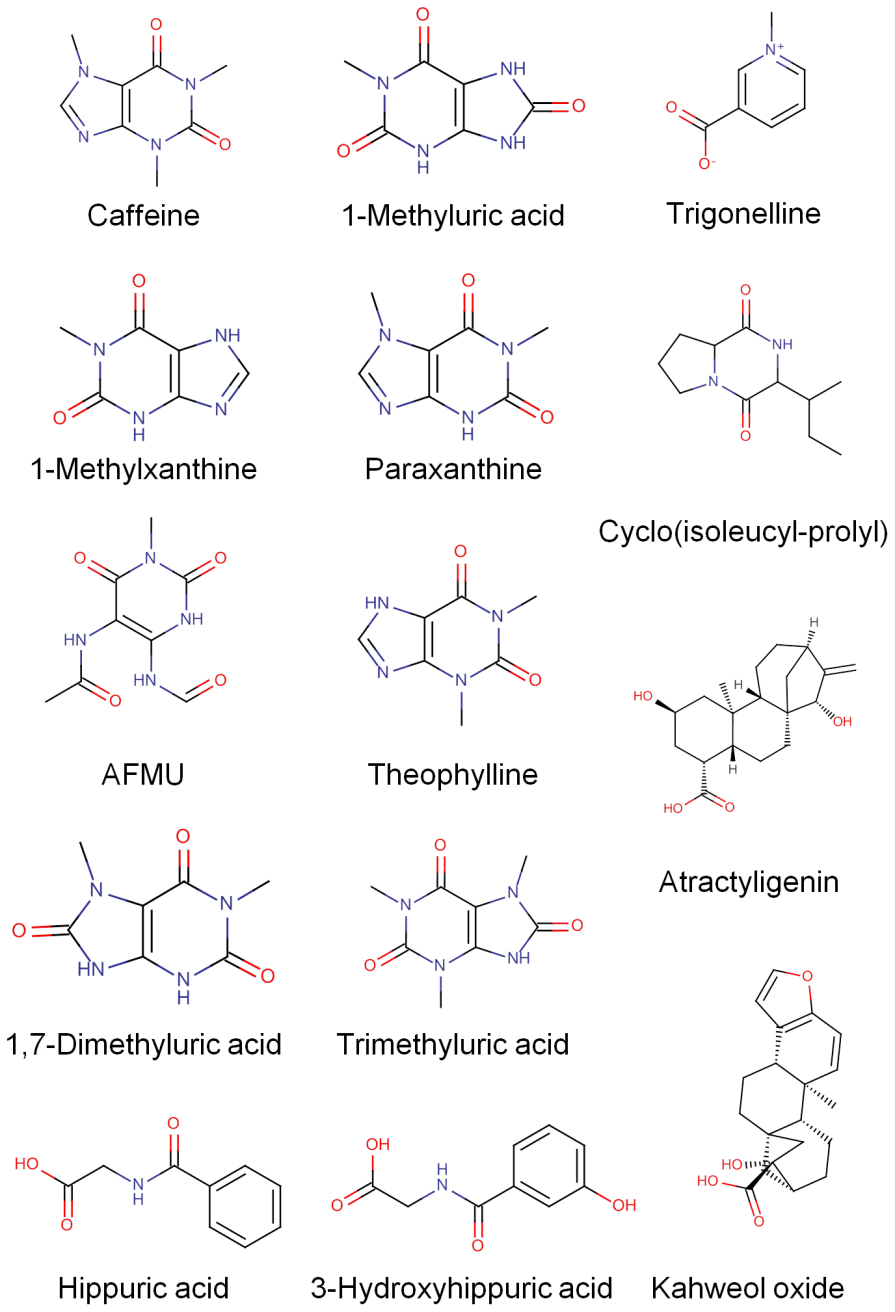
Chemical structures of some identified discriminants.

The strongest discriminating clusters are listed in Table 1 by ascending ANOVA BH p-value of the most significant ion in the cluster. Many of these were easily identified as caffeine metabolites (**Figure 2**), based on the comparison of accurate mass, RT and fragmentation spectra of unknown features and commercial standards. Among these were paraxanthine, a glucuronide of either paraxanthine or theophylline, 1-methylxanthine, 1-methyluric acid, 1,7-dimethyluric acid, 1,3 or 3,7 dimethyluric acid, 1,3,7-trimethyluric acid as well as 5-acetylamino-6-formylamino-3-methyluracil (AFMU). Of these, 1-methylxanthine (p-value = 8.51×10^−7^, VIP = 2.71) contributed most strongly to the discrimination and was the third strongest discriminant found in the study overall, with a 5-fold greater mean intensity in the high consumers than in the low. 1,7-dimethyluric acid was similarly elevated in the high consumers (p-value = 8.51×10^−7^, VIP = 2.72, 3.3-fold difference in mean intensity between the two groups).

**Table 1:** The strongest contributors to the discrimination of low and high coffee consumers.

							Highest ranked ion in cluster			
Assigned identity	Molecular formula	Theoretical m/z	Calculated m/z	Error (ppm)	Cluster ions (p-value rank)^a^	RT (min)	ANOVA BH p-value	PLS-DA VIP	Mean intensity high consumers/low consumers (ratio)	ROC curve AUC (95% CI)
Atractyligenin glucuronide^c^	C25H36O10	496.2301	496.2299	0.37	**n495.226 (1)**, p520.225 (2), p519.221 (3), p321.194 (5), p322.202 (6), p285.187 (7), p303.195 (12), p257.191 (23), p479.199 (24), p497.156 (104)	11.3	7.47×10^−9^	2.98	80/1399 (17.5)	0.980 (0.916–1)
Cyclo(isoleucyl-prolyl)^c^	C11H18N2O2	210.1368	210.1366	1.05	**p211.146 (4)**	8.4	1.61×10^−7^	2.81	120/611 (5.1)	0.969 (0.868–1)
1-Methylxanthine^b^	C6H6N4O2	166.0491	166.0484	4.06	**n165.045 (8)**, p168.059 (31), p167.054 (84), p211.073 (59)	4.4	8.51×10^−7^	2.71	88/444 (5.1)	0.965 (0.868–1)
1,7 Dimethyluric acid^b^	C7H8N4O3	196.0596	196.0592	2.23	**p197.068 (9)**, n180.034 (17), n195.056 (18), p198.071 (19)	5.8	8.51×10^−7^	2.72	923/3045 (3.3)	0.954 (0.833–1)
Kahweol oxide glucuronide^c^	C26H32O10	504.1995	504.1987	1.63	**p329.186 (10)**, p527.193 (21)	11.4	1.32×10^−3^	2.13	210/769 (3.7)	0.952 (0.797–1)
1-Methyluric acid^b^	C6H6N4O3	182.0440	182.0434	3.23	**n181.04 (11)**, n138.033 (14), p419.016 (29), p419.016 (29), p184.056 (38)	3.6	6.48×10^−6^	2.60	197/881 (4.5)	0.917 (0.738–1)
Trigonelline^b^	C7H7NO2	137.0477	137.0473	2.72	**p138.052 (13)**	0.7	8.68×10^−6^	2.57	559/2314 (4.1)	0.928 (0.762–1)
Dimethylxanthine (Paraxanthine or Theophylline) glucuronide^c^	C13H16N4O8	356.0968	356.0968	0.03	**p357.104 (15)**, p339.095 (27)	5.3	6.75×10^−5^	2.43	93/420 (4.5)	0.892 (0.762–1)
Unknown 1	C13H13NO	199.0993	199.0993	0.17	**p200.108 (16)**	5.6	6.75×10^−5^	2.42	32/146 (4.6)	0.942 (0.833–1)
Unknown 2					**p292.158 (20)**	5.8	6.75×10^−5^	2.41	319/788 (2.5)	0.900 (0.749–1)
Unknown 3					**p243.135 (22)**	7.4	1.02×10^−4^	2.38	70/175 (2.5)	0.910 (0.762–1)
Unknown 4					**p259.165 (25)**, p281.149 (125)	9.8	2.33×10^−4^	2.31	91/191 (2.1)	0.661 (0.048–0.905)
Unknown 5					**p217.100 (26)**	6.1	4.57×10^−4^	2.25	84/182 (2.2)	0.870 (0.643–1)
Unknown 6					**p285.183 (27)**	10.1	7.45×10^−4^	2.20	89/214 (2.4)	0.891 (0.69–1)
Unknown 7					**p291.124 (30)**	11.6	8.48×10^−4^	2.18	95/157 (1.7)	0.907 (0.749–1)
AFMU^b^	C8H10N4O4	226.0702	226.0701	0.79	**p185.072 (32**), p227.079 (46), p158.065 (48)	1.5	1.32×10^−3^	2.13	170/452 (2.7)	0.828 (0.594–1)
Kahweol oxide glucuronide analogue^c^	C26H34O11	522.2101	522.2092	1.68	**p329.174 (33)**	9.7	1.32×10^−3^	2.13	68/315 (4.6)	0.871 (0.69–1)
Unknown 8					**p332.243 (34)**	9.6	1.36×10^−3^	2.13	229/416 (1.8)	0.879 (0.678–1)
Unknown 9					**p355.178 (35)**	12.4	1.99×10^−3^	2.09	82/234 (2.9)	0.871 (0.667–1)
Hippuric acid^b^	C9H9NO3	179.0595	179.0595	−0.13	**p359.127 (36)**, p105.03 (51), p77.042 (55), p180.067 (58), p106.0.38 (65), n179.057 (115)	6.4	2.36×10^−3^	2.06	105/165 (1.6)	0.796 (0.571–0.952)
Unknown 10					**p243.135 (37)**, p226.110 (63)	6.9	2.36×10^−3^	2.07	128/283 (2.2)	0.910 (0.762–1)
Unknown 11					**p330.192 (39)**	6.9	2.48×10^−3^	2.06	470/872 (1.9)	0.866 (0.667–1)
Unknown 12					**p305.113 (40)**	9.1	2.73×10^−3^	2.04	170/253 (1.5)	0.865 (0.654–1)
Unknown 13					**p411.168 (41)**	11.5	2.76×10^−3^	2.04	139/430 (3.1)	0.843 (0.654–0.989)
Unknown 14					**p406.014 (42)**	3.5	2.88×10^−3^	2.03	39/102 (2.6)	0.847 (0.642–1)
Unknown 15					**p154.048 (43)**	4.4	3.00×10^−3^	2.02	64/254 (3.9)	0.846 (0.63–1)
Unknown 16					**p637.358 (44)**	12.8	3.00×10^−3^	2.02	112/188 (1.7)	0.859 (0.69–1)
Trimethyluric acid^c^	C8H10N4O3	210.0747	210.0747	0.12	**n209.074 (45)**	6.5	3.28×10^−3^	2.01	80/243 (3.0)	0.818 (0.605–0.985)
Paraxanthine^b^	C7H8N4O2	180.0647	180.0638	4.95	**p181.071 (49)**, p182.075 (54)	5.9	3.85×10^−3^	1.98	1235/4131 (3.3)	0.898 (0.726–1)
Unknown 17					**p413.142 (50)**	7.6	3.8×10^−3^	1.99	306/432 (1.4)	0.851 (0.643–0.982)
3-hydroxyhippuric acid* b	C9H9NO4	195.0532	195.0527	2.33	**n194.050 (108)**, p121.027 (113), n150.058 (128)	5.7	2.92×10^−2^	1.63	454/901 (2.0)	0.685 (0.071–0.905)
1,3- or 3,7-dimethyluric acid* ^e^	C7H8N4O3	196.0596	196.0593	1.80	**p197.068 (78)**	5	1.43×10^−2^	1.78	312/1127 (3.6)	0.797 (0.571–1)
Caffeine* b	C8H10N4O2	194.0804	194.0799	2.36	**p195.089 (100)**	7.2	2.66×10^−2^	1.65	580/1207 (2.1)	0.793 (0.557–0.989)

a The highest ranked ion from each cluster, based on ANOVA p-value, is indicated in bold.

b Identified by comparison with authentic standard.

c Tentative identification.

*Outside the 50 most discriminating ions but included as a known coffee-derived metabolite.

Also among the most discriminating ions were non-caffeine metabolites which represented even more promising candidates for biomarkers of coffee consumption (Figure 2). The most significant of these was a large cluster eluting at 11.3 min. The parent was tentatively identified as atractyligenin glucuronide based on the accurate masses of the parent and in-source fragments observed in the mass spectra obtained with high resolution mass spectrometry analysis of urine samples. The identification was further supported by the fragmentation pattern of ions of interest in hydrolyzed and non-hydrolyzed urine samples (Supporting Information S2).

The ion *m/z* 211.146, representing the second most significant metabolite, was tentatively identified as an isomer of the diketopiperazine cyclo(leucyl-prolyl), most likely the cyclo(isoleucyl-prolyl). The fragmentation spectrum was very similar to that of the cyclo(leucyl-prolyl) standard spiked in blank urine but with a slightly shorter retention time (Supporting Information S3). Very similar fragmentation patterns have previously been reported for the two compounds extracted from roasted coffee [24]. Another discriminant strongly correlated with coffee intake was the alkaloid trigonelline (*m/z* 138.055), which was identified by comparison of exact mass and RT with the authentic standard. Two other discriminants eluting at 9.7 (C_26_H_34_O_11_) and 11.4 min (C_26_H_32_O_10_) were found to be glucuronide conjugates with very similar fragmentation spectra. The most plausible hypothesis for the cluster eluting at 11.4 min was a glucuronide of a kahweol metabolite produced by oxidation of a primary alcohol. Oxidation was the most likely biotransformation predicted by Meteor software for kahweol. The cluster eluting at 9.7 min might be a hydrated analogue that lost a water moiety during ionisation. Hippuric acid and its 3-hydroxy derivative were also correlated with coffee consumption.

The ratio of mean intensity in high consumers/mean intensity in low consumers (Table 1) varied from 1.4 for 3-hydroxyhippuric acid to 17.5 for atractyligenin glucuronide, with values around 4–5 for cyclo(isoleucyl-prolyl), kahweol oxide glucuronide, dimethylxanthine glucuronide and trigonelline. For some compounds (i.e. trigonelline, 1,7 dimethyluric acid, paraxanthine) the mean intensity in non-consumers was relatively high, suggesting other possible origins than coffee.

**Figure 3:**
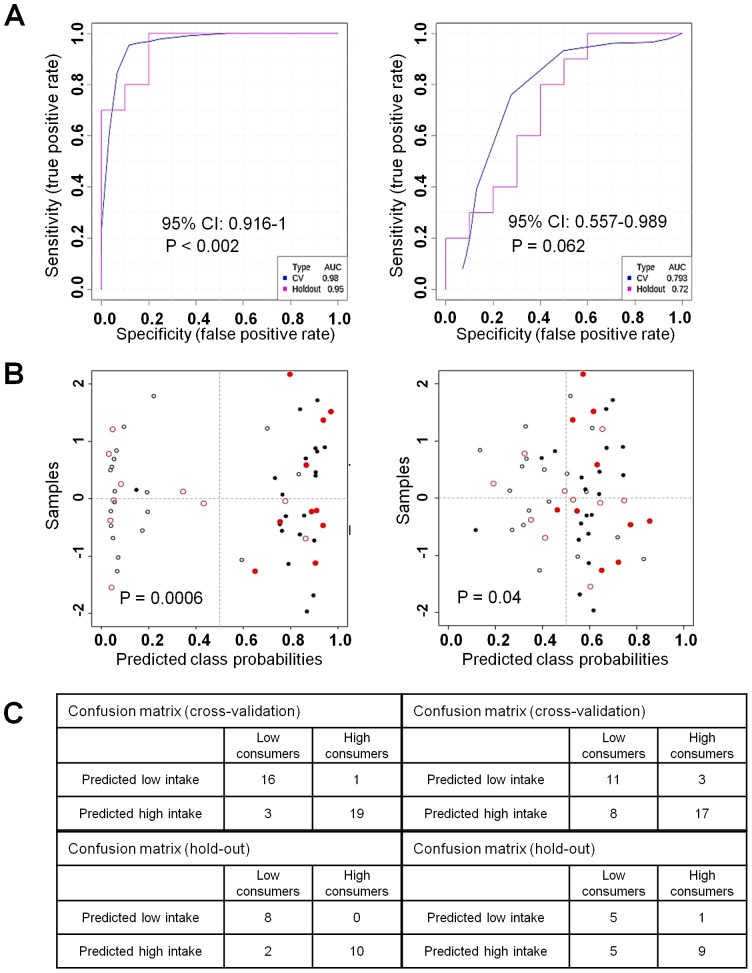
ROC curve analysis of atractyligenin glucuronide and caffeine. Data for atractyligenin glucuronide are presented in the left-hand column and data for caffeine in the right-hand column. A) Blue curves represent the training set (n = 39 subjects) and pink curves the hold-out set (n = 20 subjects). B) Probabilities of predicted belonging to the high consumer class. Training set, black plots; hold-out set, red plots; filled circles, high consumers; empty circles, low consumers. C) Confusion matrices for the two datasets.

The performance of the candidate biomarkers was assessed using ROC curves [23]. Table 1 shows the AUC and 95% CI obtained for the most important discriminants. According to the accepted classification of biomarker utility, candidate markers of AUC>0.9 are considered “excellent”, which was the case for 12 of the 33 most discriminating markers (Table 1). Sixteen others were classified as “good” (0.8–0.9), 3 as “fair” (0.7–0.8) and 2 as “poor” (0.6–0.7). It is more accurate to consider the 95% CI, which gives a spread of possible values. AFMU, for example (AUC = 0.83), could be classified as a “good” biomarker. However, the lower 95% CI limit is 0.594. Lower 95% CI limits greater than 0.8 were observed for six discriminants only: atractyligenin glucuronide, cyclo(isoleucyl-prolyl), 1-methylxanthine, 1,7-dimethyluric acid, kawheol oxide glucuronide and an unidentified discriminant (unknown1; p200.108). The performance of atractyligenin glucuronide and caffeine is illustrated in **Figure 3**. The results obtained with the validation population (10 low and 10 high coffee consumers) confirmed that atractyligenin glucuronide would be a much more effective marker of coffee consumption than caffeine (AUC 0.95 vs 0.72; Figure 3A). Furthermore, the results of the permutation test (n = 500) showed that the model based on atractyligenin glucuronide is significant (p-value<0.002), whereas the one based on caffeine is not (p-value = 0.062). The caffeine based model was able to correctly classify most high coffee consumers (Figure 3B), but misclassified half of the low-consumers, either in the training set or hold-out set. Finally, the predictive model of caffeine is sufficiently sensitive but not specific (p = 0.04), unlike the atractyligenin glucuronide model (p = 0.0006).

**Figure 4:**
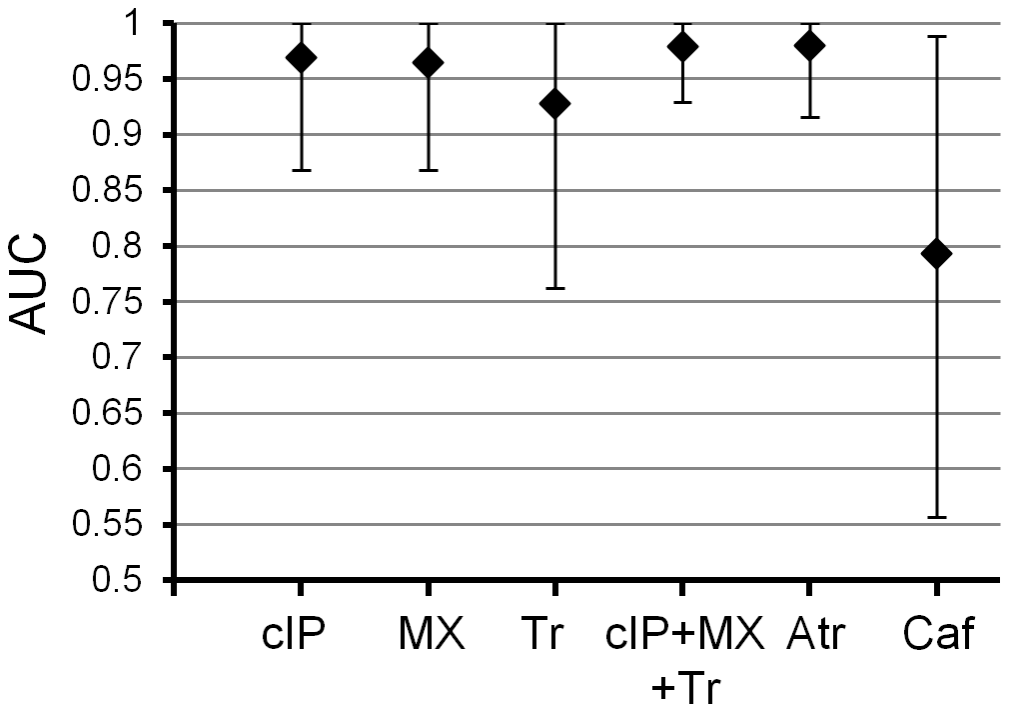
ROC curve AUCs for single and combination biomarkers. Error bars represent 95% confidence intervals. cIP, cyclo(isoleucyl-prolyl); MX, 1-methylxanthine; Tr, trigonelline; Atr, atractyligenin glucuronides; Caf, caffeine.

Since atractyligenin glucuronide is not commercially available, we tested the performance of a combination of cyclo(isoleucyl-prolyl), 1-methylxanthine and trigonelline, the three best candidate biomarkers commercially available. ROC curve analysis showed that this combination would be a more effective biomarker (better AUC and 95% CI) than any of the compounds alone (**Figure 4**).

For the three new biomarkers revealed in the study, namely atractyligenin glucuronide, cyclo(isoleucyl-prolyl) and kahweol oxide glucuronide, Pearson's correlation between their intensity in urine and the declared coffee intake of the subjects was analyzed in a SU.VI.MAX2 group of 260 subjects and showed significant positive correlations (p<0.0001, R = 0.534, 0.543 and 0.561, respectively; Supporting Information S4). Pearson's correlation coefficient was also high for 1-methylxanthine (0.508) and trigonelline (0.467) but low for caffeine (0.257), hippuric acid (0.199), and 3-hydroxyhippuric acid (0.214).

## Discussion

Urine metabolomic profiles of well-characterized groups of high and low coffee consumers from the SU.VI.MAX2 cohort were easily distinguished (Figure 1). Sensitive and specific biomarkers of coffee intake could then be searched for among the discriminant features. Some of these features corresponded to previously reported markers of intake. Caffeine, first proposed as a marker of intake some decades ago [25], was indeed a discriminant of coffee intake in the SU.VI.MAX2 cohort, although a relatively weak one (p-value = 0.0266; VIP = 1.65), and a moderately intense signal was found even in the urine of the low consumers. Some of its metabolites were better discriminants, although caffeine is found in tea, cola, energy drinks and supplements, compromising specificity for coffee intake. Also, caffeine metabolism is known to be affected by various factors, including genetic variation in the CYP1A2 gene, and caffeine clearance can vary to up to 40-fold between individuals [26]. The resulting inter-individual variation in urinary caffeine metabolites is not desirable for biomarkers of intake.

Trigonelline, another alkaloid, has previously been reported as a marker of coffee consumption in intervention participants who had consumed acute doses of coffee [27]. Trigonelline was the 7^th^ strongest discriminant between the groups in our study (p-value = 8.68×10^−6^, VIP = 2.57), and the 2^nd^ most intense discriminant in urine of high coffee consumers. However, it is also found in alfalfa sprouts, lentils, chickpeas, oats and fenugreek [28], [29] and is a plasma and urinary metabolite of niacin (vitamin B3). The trigonelline metabolite N-methylpyridinium, also reported as a marker of coffee intake [27], was not among the discriminants in the present study. Masses corresponding to cafestol and kahweol, the well-known coffee diterpenoids, were not observed among the discriminants, although a feature at [M+H]^+^ 329 was tentatively identified as a kahweol oxide glucuronide. The metabolism of cafestol and kahweol in humans is poorly documented. An intervention study in ileostomy volunteers showed that both are well absorbed in the small intestine but little is excreted as conjugates of glucuronic acid or sulfate in urine, suggesting other metabolic routes such as oxidation [30]. Oxidation of the primary alcohol of kahweol with subsequent glucuronidation of this alcohol was the first biotransformation predicted by Meteor software, supporting our tentative identification. If confirmed, kahweol oxide glucuronide may deserve further qualification as a new biomarker of coffee intake since the beverage is the only known dietary source of kahweol. It may, however, reflect only the consumption of unfiltered coffee such as expresso or Scandinavian-type boiled coffee, as brew preparations with paper filter have been shown to trap most of cafestol and kahweol in the filter [31].

Coffee is by far the greatest dietary source of hydroxycinnamic acids in human diets, and various chlorogenic acid isomers are absorbed and excreted in humans after coffee consumption [32], [33]. Previous intervention studies on polyphenols have suggested that urinary chlorogenic acids could be specific biomarkers of coffee consumption [34], [35] and the non-targeted metabolomic profiling of intervention subjects who had consumed acute doses of coffee [36] identified specific hydroxycinnamates as potential markers, of which dihydrocaffeic acid 3-sulfate and feruloylglycine might be the most promising due to their relatively long Tmax values (>4 h). Hydroxycinnamates and other phenols were detected in our study but did not contribute to the discrimination between high and low consumers. One explanation might be insufficient specificity for coffee consumption, since they are also widely consumed from fruits. Hydroxycinnamate metabolites could be useful as compliance biomarkers in controlled intervention studies but of limited use in cohort studies where subjects freely consume a variety of plant foods, and especially when only spot urines are available, since compounds with short half-life are probably not recovered in these samples. Hippuric acid and 3-hydroxyhippuric acid are end-products of the microbial catabolism of numerous polyphenols and aromatic amino acids. They have also been reported as discriminant for many physiopathological conditions or for exposures to chemical toxins in metabolomics studies [37]. Despite coffee being the richest dietary source of chlorogenic acids which are degraded to hippuric and hydroxyhippuric acids, they cannot be considered reliable biomarkers of coffee intake.

Beyond the known coffee phytochemical metabolites described above, our data-driven approach revealed some novel candidate biomarkers with high specificity and sensitivity. Atractyligenin glucuronide was the strongest discriminant in the study (p-value = 7.47×10^−9^, VIP = 2.98), and also demonstrated the greatest mean difference in intensity between high and low-consumers (17-fold). Atractyligenin is a diterpenoid whose glycoside, atractyloside, and other derivatives are present in green and roasted *Coffea arabica* beans in concentrations as high as 624 mg/kg [38], [39]. Atractyloside derivatives are also present in many plants used in ethnomedicines, but have not been reported in any other human foodstuff. Atractyloside is well known as an exceptionally specific and effective inhibitor of the ADP/ATP transport in mitochondria, able to block oxidative phosphorylation (34). Atractyligenin glucuronide has been identified in the urine of habitual coffee consumers at 3 µg/mL [40], but is proposed for the first time here as a biomarker of coffee intake. Its potential contribution to coffee health effects may also deserve further attention. The diketopiperazine cyclo(isoleucyl-prolyl) was the second most discriminating ion in the study (p-value = 1.61×10^−7^, VIP = 2.81) and thus another potential biomarker of consumption. It is known to be a bitter constituent of coffee [24]. Diketopiperazines have not previously been proposed as biomarkers of consumption, although urinary cyclo(pro-pro) and cyclo(ser-tyr) have been found to correlate with high cocoa consumption [41].

Metabolomics is essentially an exploratory approach, with some limitations. No method of profiling can cover the full chemical space of the food metabolome, and identification of unknowns is challenging [42]. Variation in study design and data processing may also lead to the discovery of different biomarkers. However, when the objective is the discovery of a biomarker of food intake, and not the comprehensive characterization of nutritional exposure following intake, it is not necessary to identify all discriminants associated with the consumption of the food, but one or a few promising candidate biomarkers only. In the present study, ROC curve analyses demonstrated that when an effective single biomarker is not found or not available as standard, a combination of several discriminants can provide a sensitive and specific biomarker.

The present work also showed that metabolomic profiling of urine samples can be applied to cohort study subjects to efficiently discover biomarkers of food consumption. A clear discrimination was achieved with relatively few subjects, but more may be required for other foods depending on their composition and patterns of consumption. The use of cohort samples can reveal more robust biomarkers than intervention studies, since they do not rely on a prescribed timeframe of sampling or exaggerated doses of the food of interest. Here, the absence of coffee hydroxycinnamate metabolites as discriminants support previous findings that many candidate biomarkers discovered in intervention studies may not be specific or robust enough for use in cross-sectional studies [16]. However, care must be taken when using cross-sectional studies for biomarker discovery since intake of other foods may correlate with that of the foods of interest. All possible dietary and metabolic origins must be carefully checked for any proposed biomarker.

Biomarker validation is a laborious process requiring dose-response and pharmacokinetics studies, attention to specificity and association with intake in various populations with different ethnic and dietary backgrounds, and consideration of the main factors affecting the relationship between the biomarker concentration and the quantity of food consumed. Our three new biomarkers were closely correlated with reported coffee intake for 260 subjects from the same SU.VI.MAX2 cohort, despite the semi-quantitative measurement of biomarker intensity. The correlations could be even more accurate if intake data were collected at the same time as urine samples in which the biomarker is quantified.

Coffee is an ideal foodstuff for which to search for biomarkers of consumption. It is rich in highly specific bioactives and its consumption is frequent in high consumers, meaning that urine concentrations of bioactives are often high. The challenge that lies ahead will be using metabolomics profiling of cohort study samples to identify biomarkers for the intake of many foods less rich in specific micro-constituents and consumed as part of complex dietary patterns.

## Supporting Information

Supporting Information S1 **Selection of low and high coffee consumers.**
(DOCX)

Supporting Information S2 **MS data supporting the tentative identification of atractyligenin glucuronide.**
(DOCX)

Supporting Information S3 **MS data supporting the tentative identification of cyclo(isoleucyl-prolyl).**
(DOCX)

Supporting Information S4 **Pearson's correlation between biomarker intensities and reported coffee intakes.**
(DOCX)
