# Supplementary material for: New Biomarkers of Coffee Consumption Identified by the Non-Targeted Metabolomic Profiling of Cohort Study Subjects
Source: PLoS One. 2014 Apr 8;9(4):e93474. doi: 10.1371/journal.pone.0093474 (PMC3979684; doi:10.1371/journal.pone.0093474)
Supplement: Supporting Information S1 — Selection of low and high coffee consumers. (DOCX) [file pone.0093474.s001.docx]

**Supporting information S1.**

Subgroups of low and high coffee consumers were selected based on distribution of coffee consumption. Of the 45 highest consumers (intake > 180 mL/d), 25 were excluded due to high consumption of another specific food, usually red wine or chocolate. A final total of 19 non-consumers and 20 high consumers (median = 290.4 mL/d) were selected for further statistical analyses.
