# Supplementary material for: New Biomarkers of Coffee Consumption Identified by the Non-Targeted Metabolomic Profiling of Cohort Study Subjects
Source: PLoS One. 2014 Apr 8;9(4):e93474. doi: 10.1371/journal.pone.0093474 (PMC3979684; doi:10.1371/journal.pone.0093474)
Supplement: Supporting Information S2 — MS data supporting the tentative identification of atractyligenin glucuronide. (DOCX) [file pone.0093474.s002.docx]

**Supporting information S2.**

Ion adducts and in-source fragments from UPLC-QToF analysis supporting the tentative identification of M = 496.2299 as atractyligenin glucuronide.

| *m/z* | RT | ESI +/- | Ion adduct or fragment |
| --- | --- | --- | --- |
| 520.225 | 11.3 | + | [M+Na]^+^ ^13^C isotope |
| 519.221 | 11.3 | + | [M+Na]^+^ |
| 497.156 | 11.3 | + | [M+H]^+^ |
| 495.226 | 11.3 | - | [M-H]^-^ |
| 479.199 | 11.3 | + | [M+H-H_2_O]^+^ |
| 322.202 | 11.3 | + | [M+H-AnhGlu]^+^ ^13^C isotope |
| 321.194 | 11.3 | + | [M+H-AnhGlu]^+^ |
| 303.195 | 11.3 | + | [M+H-AnhGlu-H_2_O]^+^ |
| 285.187 | 11.3 | + | [M+H-AnhGlu-2H_2_O]^+^ |
| 257.191 | 11.3 | + | [M+H-AnhGlu-2H_2_O-CO]^+^ |

High resolution MS data (LTQ Orbitrap) supporting the identification of atractyligenin glucuronides.

1. Extracted ion chromatogram *m/z* 495.2234 of a non-hydrolyzed urine from a high coffee consumer showing three peaks at RT 12.3, 12.99 and 13.53 min. These peaks correspond respectively to those observed at RT 10.1, 10.8 and 11.3 min with the UPLC-QTof analysis. Similarities between isotopic patterns (inset) suggest that these three peaks are different glucuronide conjugates of atractyligenin.
2. Collision-induced dissociation spectrum of *m/z* 495.2234 produces two major fragments, *m/z* 193.0369 and *m/z* 319.1947, corresponding to [M-H]^-^ of glucuronic acid and [M-H]^-^ of atractyligenin.
3. MS^3^ spectra obtained from the successive collision-induced dissociation of the ion *m/z* 495.2234 and the daughter ion *m/z* 319.1945 produces identical fragmentation patterns for the three isomers.
4. Extracted ion chromatogram *m/z* 319.1945 from the urine of a high coffee consumer after *β*-glucuronidase-sulfatase hydrolysis, showing only one peak.
5. Collision-induced dissociation spectrum of *m/z* 319.1945 in hydrolyzed urine produces similar fragments to those produced by MS^3^ spectra of non-hydrolyzed urine (C). The main fragments are derived from combined losses of H_2_O, CO, CO_2_ and HCOOH, and are consistent with the fragmentation patterns predicted by Mass Frontier^TM^ software for the proposed identification of atractyligenin glucuronide.
