# Supplementary material for: New Biomarkers of Coffee Consumption Identified by the Non-Targeted Metabolomic Profiling of Cohort Study Subjects
Source: PLoS One. 2014 Apr 8;9(4):e93474. doi: 10.1371/journal.pone.0093474 (PMC3979684; doi:10.1371/journal.pone.0093474)
Supplement: Supporting Information S3 — MS data supporting the tentative identification of cyclo(isoleucyl-prolyl). (DOCX) [file pone.0093474.s003.docx]

**Supporting information S3**.

High resolution (LTQ-Orbitrap) MS data supporting the identification *m/z* 210.1366 as cyclo(isoleucyl-prolyl).

1. MS^2^ chromatogram of *m/z* 211.1439 from a high coffee consumer urine
2. MS^2^ chromatogram of *m/z* 211.1439 from the same urine spiked with 10 µM cyclo(leucyl-prolyl).
3. MS^2^ spectrum of the compound detected as peak 1 in the spiked urine
4. MS^2^ spectrum of the standard cyclo(leucyl-prolyl) detected as peak 2 in the spiked urine
