# Supplementary material for: New Biomarkers of Coffee Consumption Identified by the Non-Targeted Metabolomic Profiling of Cohort Study Subjects
Source: PLoS One. 2014 Apr 8;9(4):e93474. doi: 10.1371/journal.pone.0093474 (PMC3979684; doi:10.1371/journal.pone.0093474)
Supplement: Supporting Information S4 — Pearson's correlation between biomarker intensities and reported coffee intakes. (DOCX) [file pone.0093474.s004.docx]

**Supporting information S4**.

Pearson’s correlation between the biomarker (ion intensity) and reported coffee intake (mL/d) for 260 SU.VI.MAX2 subjects. A) Atractyligenin glucuronide B) cyclo(isoleucyl-prolyl) and C) kahweol oxide glucuronide. R= Pearson correlation coefficient.
